# State Medical Society

**Published:** 1853-08

**Authors:** 


"STATE MEDICAL SOCIETY."

The following is a *synopsis* of the proceedings of the "State Medical Society," which convened at Davenport, on the 5th of June last. Dr. Rauch, who kindly furnished this, has also carefully prepared a full report, but for want of means in the treasury, its publication will be delayed. So soon as the annual assessment is paid in, then the report will be forthcoming. This oversight on the part of the members will doubtless be promptly and cheerfully remedied so soon as those who are delinquent will have had their attention invited to it. We do not know a member who will not at once meet his obligations to the Society, under the circumstances; and in accordance with the wish of the Recording Secretary, we have made the statement and trust that the obstacle to the publication will as early as possible be removed. We defer to a future number the few thoughts we design to submit touching the inexcusable haste of our proceedings at the annual convocations of the society.

Davenport, June 8, 1853.

Society convened according to adjournment at 10 o'clock, A. M., and was called to order by the President, Dr. Elbert.

The Recording Secretary being absent Dr. C. G. Blood, on motion was appointed Recording Secretary pro tem.

The proceedings of the last annual meeting were then read, also the Constitution and By-Laws.

There being a vacancy in the Board of Censors, Dr. Witherwax on motion was appointed Censor pro tem.

On motion of Dr. McGugin, a committee of three was appointed to nominate officers for the ensuing year. The President appointed Drs. McGugin, Siveter and Blood said Committee.

The Board of Censors recommended Drs. Arnold, Wainwright and Morgan, for membership, who, upon complying with the requisitions of the Constitution, were duly declared members of the Society.

On motion of Dr. Ford, a committee of three was appointed to nominate delegates to the American Medical Association, Drs. Ford, Wainwright and Blood were appointed.

On motion the Society adjourned until 2 o'clock, P. M.

AFTERNOON SESSION, 2, P. M.

Society was called to order by the President.

The Annual Address by the President, was then delivered.

The Board of Censors recommended for membership Drs. C. C. Parry, Ignatius Langer, George Reeder and C. O. Waters, who complying with the usual requisitions were duly declared members of the Society.

The Nominating Committee reported the following for officers for the ensuing year:

| | |
|---|---|
| Dr. J. M. WITHERWAX, | President. |
| Dr. T. SIVETER, | 1st Vice " |
| Dr. GEO. REEDER, | 2d Vice " |
| Dr. J. H. RAUCH, | Recording Secretary. |
| Dr. D. L. McGUGIN, | Corresponding Secretary. |
| Dr. G. R. HENRY, | Treasurer. |
| Dr. ASA MORGAN, | Librarian. |

*Censors.*

Dr. J. D. ELBERT, Dr. J. F. HENRY, Dr. C. O. WATERS, Dr. J. C. HUGHES, Dr. J. F. SANFORD, Dr. WM. CRAIG, Dr. I LANGER.

The Officers nominated were then ballotted for, and duly elected.

On motion, Dr. Elbert conducted Dr. Witherwax, the President elect to the chair, who upon taking it returned thanks for the honor conferred upon him, in a brief and appropriate manner.

The committee to report delegates to the American Medical Association, reported as follows: Drs. J. D. Elbert, Geo. Reeder, G. R. Henry, J. M. Witherwax, T. Siveter and J. T. Huey.

On motion the report was adopted.

Report of Dr. G. R. Henry, Chairman of Committee on the Second District was read.

Report of Committee on Surgery, by Drs. Elbert and Sanford was then read.

Report of Committee on Obstetrics and Diseases of Females and Children by Dr. McGugin was also read.

Several interesting cases were reported by Dr. Langer, which after an interesting discussion, on motion with the above reports was ordered to be printed in the proceedings.

On motion of Dr. Rauch the Standing Committees were continued.

Dr. McGugin offered the following resolution which was adopted.

Resolved, That we have heard with profound regret of the recent death of Prof. G. W. Richards of Dubuque, and from the prominent position which he has occupied in the profession, the devotion he has ever exhibited in behalf of science, the efficiency of his character and usefulness of his past life, we are called upon to give an expression of our regret for the loss which the profession has sustained in his death, and in this manner to offer to his family and friends our sympathies and condolence for their irreparable loss.

Dr. Elbert offered the following resolution, which was adopted.

Resolved, That Prof. McGugin be requested to address a suitable letter of condolence to the family and friends of Prof. G. W. Richards, with a copy of the above resolution.

On motion the Society adjourned until 7½, P. M.

Evening Session.

Society called to order by the President, at 7½ P. M.

The Board of Censors recommended Dr. Rosseau, for membership, who complying with the Constitution, was duly declared a member of the Society.

Dr. Rauch offered the following resolution, which was adopted:

Resolved, That the proceedings of last year be published with those of this year, and the Recording Secretary and Treasurer be authorized to levy such a contribution from each member, as will defray the expenses incurred in so doing.

On motion of Dr. Elbert the Recording Secretary was appointed a committee of publication.

Dr. Craig offered the following resolution, which was adopted:

Resolved, That the thanks of the Society be tendered to the physicians of Davenport, for their marked attention and kindness during the meeting of the Society.

On motion of Dr. Reeder the Society adjourned to meet at Muscatine on the 2nd Monday in June, 1854, at 2 o'clock, P. M.

